# The Competitive Loss of Cerebellar Granule and Purkinje Cells Driven by X-Linked Mosaicism in a Female Mouse Model of CASK-Related Disorders

**DOI:** 10.3390/cells14100735

**Published:** 2025-05-17

**Authors:** Takuma Mori, Mengyun Zhou, Ken Kunugitani, Taichi Akatsuka, Yukina Yoshida, Emi Kouyama-Suzuki, Shin Kobayashi, Yoshinori Shirai, Katsuhiko Tabuchi

**Affiliations:** 1 Department of Molecular and Cellular Physiology, Shinshu University School of Medicine, Matsumoto 390-8621, Japan or mori@inst-hsc.jp (T.M.); 20hm124d@shinshu-u.ac.jp (M.Z.); kunuken846@gmail.com (K.K.); 19m001j@shinshu-u.ac.jp (T.A.); 21s5029a@shinshu-u.ac.jp (Y.Y.); emi_suzuki@shinshu-u.ac.jp (E.K.-S.); yoshirai@shinshu-u.ac.jp (Y.S.); 2 Department of Neuroinnovation, Institute for Biomedical Sciences, Interdisciplinary Cluster for Cutting Edge Research, Shinshu University, Matsumoto 390-8621, Japan; 3 Cellular and Molecular Biotechnology Research Institute, National Institute of Advanced Industrial Science and Technology, Tokyo 135-0064, Japan; kobayashi.shin@aist.go.jp

**Keywords:** CASK, cell autonomous, MICPCH, cerebellum, X-chromosome inactivation, cell competition

## Abstract

CASK-related disorders are a form of female-restricted intellectual disabilities associated with cerebellar and pontine hypoplasia. The *CASK* gene is regulated by X-chromosome inactivation, which results in a mosaic distribution of CASK-expressing and CASK-deficient neurons in the female brain. This mosaic distribution is believed to play a key role in the pathophysiology of X-linked neurological disorders; however, the detailed brain structure has not been extensively characterized. In this study, we used CASK heterozygous knockout (CASK-hKO) mice combined with X-linked GFP reporter mice to investigate motor abilities and the distribution of CASK-expressing cells in the brains of female CASK-hKO mice. The CASK-hKO mice exhibited motor deficits and cerebellar hypoplasia similar to those observed in patients with CASK-related disorders. Interestingly, although half of the cerebellar granule cells were CASK-negative during early postnatal development, almost all Purkinje cells and cerebellar granule cells were CASK-positive in adulthood, suggesting that CASK expression may determine the survival of cerebellar granule cells during postnatal development. We also analyzed CASK-hypomorphic mice, which express 50% less CASK than wild-type mice, and compared hemizygous males and heterozygous females. The CASK-hypomorphic heterozygous females displayed a thinner cerebellar cortex and a higher probability of CASK-positive granule cells in CASK-hKO females, suggesting that the survival of cerebellar granule cells is regulated by a combination of cell-autonomous and cell-competitive mechanisms between CASK-expressing and CASK-deficient cells, which are generated by X-chromosome inactivation. These findings provide new insights into the relationship between the mosaic distribution of cells established by X-chromosome inactivation and the pathophysiology of CASK-related disorders.

## 1. Introduction

Calcium-dependent serine/threonine kinase (CASK) is a member of the membrane-associated guanylate kinase (MAGUK) proteins family and was initially identified as a binding partner of neurexin [1]. MAGUK proteins are generally localized at synaptic terminals and regulate the trafficking and targeting of ion channels and synaptic proteins [2,3,4,5,6,7]. The CASK protein contains five functional domains, a catalytic serine/threonine kinase (also known as calmodulin kinase, CaMK) domain, two Lin2/Lin7 (L27.1 and L27.2, LIN) domains, a PSD-95/disc large/ZO-1 (PDZ) domain, an Src Homology 3 (SH3) domain, and a guanylate kinase (GuK) domain [8].

An association between CASK and clinical phenotypes was first described by Dimitratos et al., who identified that the *CASK* gene is located on the locus of X-linked optic atrophy [9]. Hayashi et al. identified the *CASK* gene as a causative gene of neurological disorders, such as X-linked intellectual disability [10]. Later, some reports further implicated CASK in other neurological disorders, including congenital nystagmus, developmental epileptic encephalopathy, and FG syndrome [11,12,13,14,15,16,17]. One of the common neurological phenotypes of CASK-associated disorders is microcephaly with pontine and cerebellar hypoplasia (MICPCH) syndrome, first reported by Najm et al. [11]. After this first report, MICPCH has been commonly observed in approximately 80% of patients with mutations of the *CASK* gene [11,18]. In relation to the gene mutation patterns of the *CASK* gene and the occurrence of MICPCH, over 80% of the patients were females and almost all the mutations were nonsense mutations such as gain of stop codon and frameshift mutations [8]. On the other hand, other clinical phenotypes such as intellectual disability or ophthalmological disorders were observed in both males and females with missense mutations. These findings indicate that MICPCH may be caused by the loss-of-function of the *CASK* gene.

MICPCH was also replicated in animal models of CASK-associated disorders. The first report of MICPCH syndrome demonstrated an atrophy of cerebellar cortex in a floxed CASK male mouse with thinner molecular and granular layers of the cerebellum [11]. In this model, the CASK protein was shown to be expressed at approximately 30%, probably because inserted LoxP sequences disturbed the transcription and/or translation of the *CASK* gene [19]. Therefore, the floxed CASK mouse can be considered as a “hypomorphic” model of CASK-associated disorders. Another animal model of MICPCH is a hetero-knockout female mouse of the *CASK* gene [20,21], where the expression of the *CASK* gene is regulated by X-chromosome inactivation (XCI). These studies have reported cerebellar atrophy in the CASK knockdown/hetero-knockout mice, but there is a contradiction among these studies. An in vitro study using primary culture of cerebellar granule cells reported that the early knockout of the *CASK* gene resulted in cell death [22], supporting the notion of cell-autonomous regulation of cerebellar granule cell survival. In contrast, cell-specific knockout of the *CASK* gene from cerebellar granule or Purkinje cells did not lead to significant shrinkage of the cerebellum in vivo [20], suggesting that a non-cell autonomous mechanism may contribute to the survival of cerebellar neurons. Consequently, the precise impact of *CASK* gene deficiency on cerebellar development in female patients with CASK-related disorders remains unclear. One possible explanation for these discrepancies could involve a cell competition mechanism since most genes on the X-chromosome, such as CASK, are regulated by XCI, and it is hypothesized that XCI might influence the pathological phenotype of X-linked disorders in females [23,24]. To date, there have been no studies investigating how CASK-expressing and CASK-non-expressing cells are distributed throughout a mouse body.

We have previously sampled a part of neocortical neurons and genotyped them using single-cell RT-PCR [21]. That study revealed that the cerebral neocortex contained mosaic distribution of CASK-expressing and non-expressing cells, but cerebellar cells were not examined. In this study, we combined GFP reporter mice that can visualize XCI patterns to analyze the distribution of cells throughout the brains of CASK-mutant mouse models and found that the presence or absence of CASK expression determines the survival of cerebellar cells. Furthermore, in order to clarify the effect of the mosaicism of two types of cells with different CASK expression levels on cell survival, CASK-hypomorphic mice with reduced CASK expression were analyzed. As a result, it was found that the cell survival rate was lower in mice with a mixture of cells with reduced CASK expression and cells with normal CASK expression than in mice with reduced CASK expression in all cells. Thus, it was suggested that there is a cell-autonomous and competitive mechanism in cerebellar hypoplasia caused by the *CASK* gene.

## 2. Materials and Methods

### 2.1. Animal Housing, Husbandry, and Welfare

All animals (total 62 mice) were group-housed, maintaining a 12:12 light–dark cycle (lighting from 09:00 to 21:00) with food and water ad libitum. The room temperature was maintained at 23 ± 2 °C. All procedures of animal experiments were reviewed by the Committee for Animal Experiments and were finally approved by the president of Shinshu University (Approved number, 023097, date of approval, 9 January 2024). The methods were carried out in accordance with the Regulations for Animal Experimentation of Shinshu University.

### 2.2. Mouse Models

#### 2.2.1. CASK-Knockout Mice

We used CASK-knockout (Cask-KO) mice, which were used in our previous study [21]. The *CASK*-floxed mice were generated by introducing two loxP sequences at the ends of the exon1 of the mouse *CASK* gene [19]. The expression level of the protein in the brain of the *CASK*-floxed mice has been reported to be approximately 30% of the CASK wild-type (CASK-WT) mice [19], and these mice showed cerebellar atrophy, which is a symptom of CASK-associated disorders [11]. CASK-KO mice were obtained by crossing female mice carrying floxed CASK (B6;129-Cask^tm1Sud^/J, JAX Stock #006382, RRID:IMSR_JAX:006382) [19] and ZP3-Cre (C57BL/6-Tg(Zp3-cre)93Knw/J, JAX Stock #003651, RRID:IMSR_JAX:003651) [25], where Cre gene expression is driven in ovum by the regulation of ZP3 promoter. To overcome the higher lethality of the heterozygous CASK-KO mice in previous studies [19,21], we maintain the CASK-floxed mice under the hybrid background of C57BL/6J and SV129. The genotype of the CASK-floxed allele, CASK-knockout allele, and ZP3-Cre allele was determined using genomic PCR using the primers listed in Table 1.

#### 2.2.2. GFP Reporter Mice for Visualization of X-Chromosome Inactivation

To visualize the pattern of X-chromosome inactivation in brain tissues, we crossed the CASK-flox/ZP3-Cre female with male mice carrying a CAG-GFP gene cassette on the hypoxanthine-guanine phosphoribosyl transferase (HPRT) gene locus of the mouse X chromosome (B6;129-Hprt<tm1(CAG-eGFP-NLS)Koba>, RBRC09532, RIKEN BioResource Research Center, Saitama, Japan) [26]. The genotype of the mouse was determined by genomic PCR using primers on Table 1 and/or by checking GFP expression using a handy blue LED with an orange filter (LEDGFP-3WOF, OptoCode Inc., Tokyo, Japan).

To obtain CASK/HPRT-GFP mice, male hemizygous HPRT-GFP mice were mated with CASK-flox/ZP3-Cre female mice to generate offspring (CASK-hKO/HPRT-GFP and CASK-WT/HPRT-GFP females) for this study. To obtain CASK-flox mice as a CASK-hypomorphic model, CASK-flox-heterozygote female mice were mated with HPRT-GFP male mice. We obtained wild-type and fCASK-hemizygote males, and CASK-wild-type/HPRT-GFP and fCASK-heterozygote/HPRT-GFP females from this combination.

### 2.3. Behavioral Assessment

All the behavioral tests were undertaken by experimenters who were blind to the genotype of the subjects. Litters of heterozygote and wild-type female mice were used in the behavioral studies. Some mice, probably heterozygote knockout, showed immobility in the home cage and they were excluded from the analysis.

#### 2.3.1. Open Field Test

Mice were placed in a square 50 cm × 50 cm × 40 cm open field arena and were allowed to move freely for 20 min. The behaviors of the mice were recorded using a USB webcam (HD Webcam C615; Logicool, Tokyo, Japan) at 30 frames per second (fps). The location of the mice was extracted from the movie using idTracker software (version of 29 April 2014) [27]. The extracted data were further analyzed using a custom-made program used in our previous study [28,29]. The distance traveled in a 10 min’ duration and the percentage of the time spent in the center area (a center square of 25 cm × 25 cm) were measured in this study. We analyzed eight CASK-hKO and CASK-WT mice (aged 2 to 4 months-old) in this experiment.

#### 2.3.2. Hind-Limb Clasping Test

To examine the hind-limb clasping score, the mouse was held by the tail 20 cm above and allowed to move its limbs for 20 s. If the hindlimbs were splayed outward, away from the abdomen, we would give a score of 0. If one or both hindlimbs were partially retracted toward the abdomen, we would give a score of 1. If both hindlimbs were retracted and touched to the abdomen, we would give a score of 2 [30]. We analyzed eight CASK-hKO and CASK-WT mice (aged 2 to 4 months-old) in this experiment.

#### 2.3.3. Wire Hang Test

The mouse was first placed on a metal lid (25 cm × 25 cm), 1 cm square of mesh was used to lightly shake the lid to make the mouse grip the wires, and then turned the lid was turned upside down. The lid was placed on the test box (20 cm × 20 cm × 40 cm), which was filled with >10 cm thickness of soft wood chips. The mouse behavior was recorded with the USB webcam for 10 min. The latency until the mouse fell was analyzed. If the mouse did not fall in 10 min, the time of the mouse was recorded as 600 s. We analyzed eight CASK-hKO and CASK-WT mice (aged 2 to 4 months-old) in this experiment.

#### 2.3.4. Rotarod Test

We use a rotarod apparatus (O’HARA & Co., Ltd., Tokyo, Japan). The starting speed was set at 4 rotation per minute (rpm) and the rod was accelerated up to 40 rpm in 5 min. The maximal speed was maintained for a minute. We measured the time duration before the mouse fell off the rod. If the mouse ran for the full duration without falling, the score of the mouse was defined as 360 s. After each test, we returned the mouse to its home cage and gave it a period there which was longer than the time on the last rotarod trial. We sanitized the rotarod apparatus with 70% ethanol every time after each trial. The rotarod test consisted of three trials per day for four consecutive days. We analyzed eight CASK-hKO and CASK-WT mice (aged 2 to 4 months-old) in this experiment.

### 2.4. Histological Analysis

#### 2.4.1. Immunohistochemistry

Under deep anesthesia, mice were perfused transcardially with ice-cold phosphate-buffered saline (PBS, pH 7.4), followed by 4% paraformaldehyde (Nacalai tesque, Kyoto, Japan) in PBS. The brain was extracted from the skull and put into 30% sucrose in PBS until it sank. After trimming the brain, 50-μm-thick coronal or sagittal sections were prepared with a sliding microtome (REM-700, Yamato Kohki Industrial, Saitama, Japan). The sections were washed with PBS, blocked with PBS containing 1% bovine serum albumin, 0.1% Triton-X100 (Fujifilm Wako Pure chemical, Osaka, Japan), and 10% normal donkey serum, and incubated with primary antibodies (Table 2). After overnight incubation with primary antibodies, the brain sections were washed with PBS containing 0.1% Triton-X100 and incubated with fluorophore-conjugated secondary antibodies for 2–3 h at room temperature. After further washing with PBS, brain sections were mounted on a slide glass, counterstained with DAPI (Tokyo Kasei, Tokyo, Japan), and coverslipped.

Fluorescence images were captured with an all-in-one fluorescent microscope (BZ-X800, Keyence, Osaka, Japan) and a confocal laser-scanning microscope (TCS SP8; Leica Microsystems, Wetzlar, Germany), both equipped with 4×, 10×, and 20× objective lenses. For the quantitative counting of cerebellar granule cells, hippocampal CA1 pyramidal cells, and doublecortin (DCX)-positive cells, we also used digital zoom (1.5× to 3×) [31]. The images were analyzed by using ImageJ (FIJI, https://fiji.sc, accessed on 9 January 2024, RRID: SCR_002285) and a custom program on R (v4.1.2; R Core Team 2021) [32]. The total number of animals is indicated in the legend of each figure and in the results.

#### 2.4.2. Estimation of Survival Rate of the CASK-Negative Neurons

The survival rate of GFP-negative cells was estimated using the proportion of GFP-positive cells obtained by the microscopic observation. The proportion of GFP-positive cells within the total cell population was designated as P_GFP+_, and the survival rate of CASK (GFP)-negative cells P_survival_ was defined as follows. First, both P_GFP+_ and P_survival_ are real numbers within the range (0,1) These values were calculated under the assumption that GFP-positive and GFP-negative cells are produced in equal numbers during the early stages of cell division and that GFP-positive cells remain present throughout the developmental stage (Supplementary Figure S1). The relationship between these variables is described by the following equation:

$$P_{GFP+}=\frac{1}{1+P_{survival}}$$

By rearranging this equation, P_survival_ can be calculated as follows:

$$P_{survival}=\frac{1}{P_{GFP+}}-1$$

This transformed equation was applied to estimate P_survival_ from the experimentally observed value of P_GFP+_.

### 2.5. Immuno-Blotting

Brain homogenates were prepared from the cerebellum of adult male mice (postnatal weeks 7–11), 3 WT mice and 3 floxed CASK mice, independently with homogenization buffer (0.32 M sucrose, 1 mM NaHCO_3_, 1 mM MgCl_2_, and 0.5 mM CaCl_2_ containing protease inhibitor cocktail (Nacalai tesque, Kyoto, Japan)) using a motor-operated Teflon/glass homogenizer (Yamamoto-Seisakusyo, Kyoto, Japan). The protein concentration of brain lysate was quantified by BioRad Protein Assay System (BioRad, Hercules, CA, USA). A total of 20 μg protein (for CASK) or 0.5 μg protein (for β-Actin) was subjected to SDS-PAGE (7.5% Laemmli) and electro-blotted onto Immobilon-P PVDF membrane (Millipore, Burlington, MA, USA). Western blotting was carried out using primary antibodies, an anti-CASK mouse monoclonal antibody (NeuroMab, Davis, CA, USA, K56A/50, RRID: AB_2877188, 1/2000 dilution), and an anti-β-Actin mouse monoclonal antibody (M177-3, 1/5000 dilution, MBL, Tokyo, Japan), and was followed by a secondary antibody, an anti-mouse IgG HRP-conjugated antibody (1706516, RRID:AB_11125547, 1/5000 dilution, BioRad, Hercules, CA, USA). The chemiluminescence signals were visualized using LuminateForte (Millipore, Burlington, MA, USA) by ChemiDoc Touch (BioRad, Hercules, CA, USA). The intensity of the band was analyzed using ImageJ (FIJI, https://fiji.sc, RRID: SCR_002285).

### 2.6. Statistical Analysis

Sample sizes were determined based on established practices and our previous experience in respective assays. The number of independent samples (e.g., neurons) is indicated on the graphs and the number of animals is indicated in the figure legends. All values are represented as the average of independent experiments ± SEM. The variance among the analyzed samples was similar. After normality was assessed by a Shapiro–Wilk test, statistical significance was determined by Student’s *t*-test. Statistical analysis was performed by custom-written R scripts and Prism 8.4 (GraphPad Software Inc., Boston, MA, USA, RRID:SCR_002798). Statistical significance is indicated by asterisks (* *p* < 0.05, ** *p* < 0.01, and *** *p* < 0.001). All data are expressed as means ± SEM.

## 3. Results

**Figure 1:**
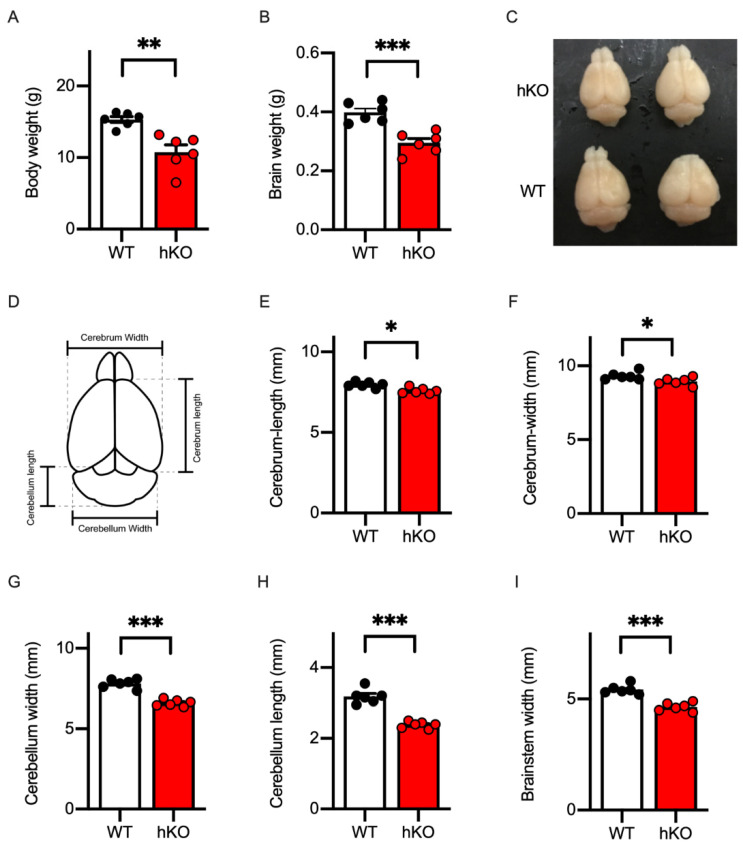
Microcephaly with cerebellar hypoplasia was replicated in CASK-heterozygote knockout (hKO) female mice. (**A**) Body weight was decreased in CASK-hKO (*n* = 6) mice in adulthood (2 months-old), compared to wildtype (WT) (*n* = 6). (**B**) Brain weight was also decreased in the CASK-hKO mice compared to WT mice. (**C**) Brains of CASK-hKO mice appeared smaller than those of CASK-WT mice. (**D**) Parameters of the brain morphology analyzed in this study are indicated. (**E**) The length of the cerebral cortex along the anterior–posterior axis was shorter in the CASK-hKO mice than in CASK-WT mice. (**F**) The width of the cerebral cortex was also shorter in the CASK-hKO mice. (G and H) The length (**G**) and the width (**H**) of the cerebellum were decreased in the CASK-hKO mice. (**I**) The width of the brainstem of the CASK-hKO mice was smaller than that of CASK-WT mice. Each dot indicates the value of an individual animal. Bars and error bars indicate the mean and the standard error. Asterisks represent statistically significant differences evaluated by Student’s *t*-test (* *p* < 0.05, ** *p* < 0.01, *** *p* < 0.001).

Patients with CASK-related disorders were reported to grow with a delay [33]. We and others reported that female CASK-hKO mice exhibited slow growth [20,21]. Consistent with the previous observations, the body weight of CASK-hKO mice was lower than those of female WT mice (Figure 1A: 10.76 ± 0.10 g of hKO vs. 15.32 ± 0.51 g of WT, *p* = 0.0018, *t*-test) and their brains were also smaller and lighter than WT mice (Figure 1B,C: 0.40 ± 0.013 g of WT vs. 0.30 ± 0.015 g of hKO, *p* = 0.0004, *t*-test). To quantify the microcephaly of the CASK-hKO mice, we measured the sizes of the cerebrum at a macroscopic level (Figure 1D). The length of the cerebrum along the anterior–posterior axis (Figure 1E) and the width along the medial–lateral axis (Figure 1F) were smaller in CASK-hKO mice than in CASK-WT mice (length: 7.97 ± 0.071 mm of WT vs. 7.59 ± 0.076 mm of hKO, *p* = 0.0045, *t*-test; width: 9.32 ± 0.11 mm of WT vs. 8.94 ± 0.11 mm of hKO, *p* = 0.0330, *t*-test). One of the prominent morphological phenotypes of CASK-related disorders is the pontine and cerebellar hypoplasia [8]. We measured the length and width of the cerebellum and the width of the brainstem of mice (Figure 1D). The size of the cerebellum of CASK-hKO mice significantly decreased in the length (Figure 1G: 3.18 ± 0.083 mm of WT vs. 2.38 ± 0.038 mm of hKO, *p* < 0.001, *t*-test) and width (Figure 1H: 7.80 ± 0.12 mm of WT vs. 6.60 ± 0.084 mm of hKO, *p* < 0.001, *t*-test), as well as in the width of the brainstem (Figure 1I: 5.43 ± 0.085 mm of WT vs. 4.65 ± 0.076 mm of hKO, *p* < 0.001, *t*-test). Thus, the CASK-hKO female mice replicated the key MICPCH phenotypes observed in human patients to a certain extent.

**Figure 2:**
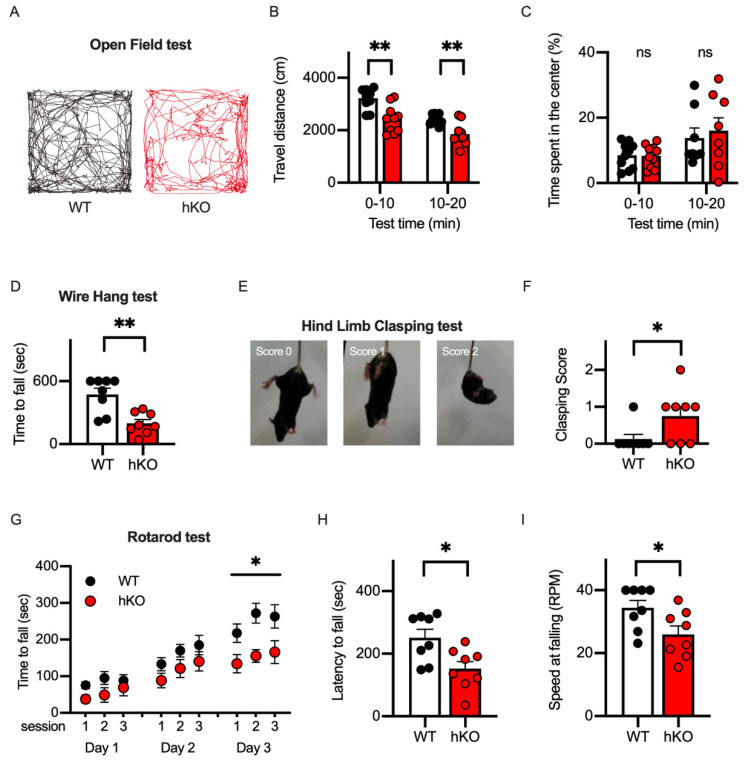
Motor ability was altered in CASK-hKO (heterozygote knockout) female mice. (**A**) Trajectories during 20 min open field test are drawn. Trajectory of a WT (wildtype) mouse is shown in black (left) and that of a hKO mouse was shown in red (right). (**B**) Travel distance during each 10 min duration decreased in hKO mice (n = 8) compared to WT mice (n = 8). (**C**) Time spent in the center area is not different between WT and hKO mice (ns: not significantly different). (**D**) Time to fall during the wire hang test decreased in hKO mice. (**E**) Representative images indicating different degrees of hind-limb clasping of a mouse. (**F**) The clasping score is higher in hKO mice than in WT mice. (**G**) Motor performance examined with a rotarod test improved more in WT mice than in hKO mice. (**H**,**I**) Latency to fall (**H**) and maximum speed of the rotation until the mouse fell (**I**) decreased in hKO mice. Each dot indicates the value of an individual animal. Bars and error bars indicate the mean and the standard error. Asterisks represent statistically significant differences evaluated by Student’s *t*-test (* *p* < 0.05, ** *p* < 0.01).

Developmental delays in motor skills have been reported as one of the most common pathological phenotypes in CASK-related disorders [8]. To examine motor abilities of the CASK-hKO female mice, we first conducted the open field test and measured the total travel distance and the time duration in the center area of the arena to evaluate the basic walking skills and the extent of the anxiety levels, respectively. Both WT and hKO mice explored the entire regions of the open field arena (50 cm × 50 cm) in the 20 min test (Figure 2A). The travel distance of CASK-hKO mice was decreased compared to the that of WT mice in the first and second halves (Figure 2B: 3222 ± 123.1 cm in WT vs. 2426 ± 165.6 cm in hKO during first 10 min, *p* = 0.0012; 2404 ± 58.8 cm in WT vs. 1854 ± 133.2 cm in hKO during 10–20 min, *p* = 0.0014).

The decrease in the travel distance could be explained by the motor deficits or the elevated anxiety level of the animal. Therefore, we examined the anxiety level of the animals by measuring the change in the travel distance between the first and second halves and the duration spent in the center region of the arena. Both WT and hKO mice showed that the travel distance decreased from the first half to the second half (Figure 2B), suggesting that both mice habituated the novel environment (the open field arena) to the same extent. The time spent in the center region was not different between WT and hKO mice (Figure 2C: 8.43 ± 1.25% in WT vs. 7.60 ± 1.20% in hKO during first 10 min, *p* = 0.6377; 13.54 ± 2.42% in WT vs. 14.48 ± 3.37% in hKO during 10-20 min, *p* = 0.8228). When these results are taken together, the CASK-hKO mice may exhibit motor deficits without an associated increase in anxiety.

We next employed a wire-hanging test and a hind-limb clasping test to the evaluate neuromuscular function and the motor-coordination of the CASK-hKO mice [34]. We measured the latency until the mouse fell from the wired grid and observed that the CASK-hKO mice fell after a shorter duration compared to the WT mice (Figure 2D: 474.0 ± 57.99 s of WT vs. 197.4 ± 36.57 s of hKO, *p* = 0.0012, *t*-test). The score of the hind-limb clasping test (Figure 2E) was also higher in the CASK-hKO mice compared with the WT mice (Figure 2F). The cerebellum has been studied in relation to the motor learning in both rodents and humans [35,36]. To assess the ability of the motor learning, we proceeded with the rotarod test (Figure 2G) and observed decreases in the latency to fall from the rotating rod (Figure 2H: 251.1 ± 26.7 s in WT vs. 152.0 ± 23.1 s in hKO, *p* = 0.0138) and in the final speed at the time of falling (Figure 2I: 34.4 ± 2.4 rpm in WT vs. 25.99 ± 2.6 rpm in hKO, *p* = 0.0327). All these results indicate that motor functions are altered in CASK-hKO mice, which is similar to clinical cases [33,37].

**Figure 3:**
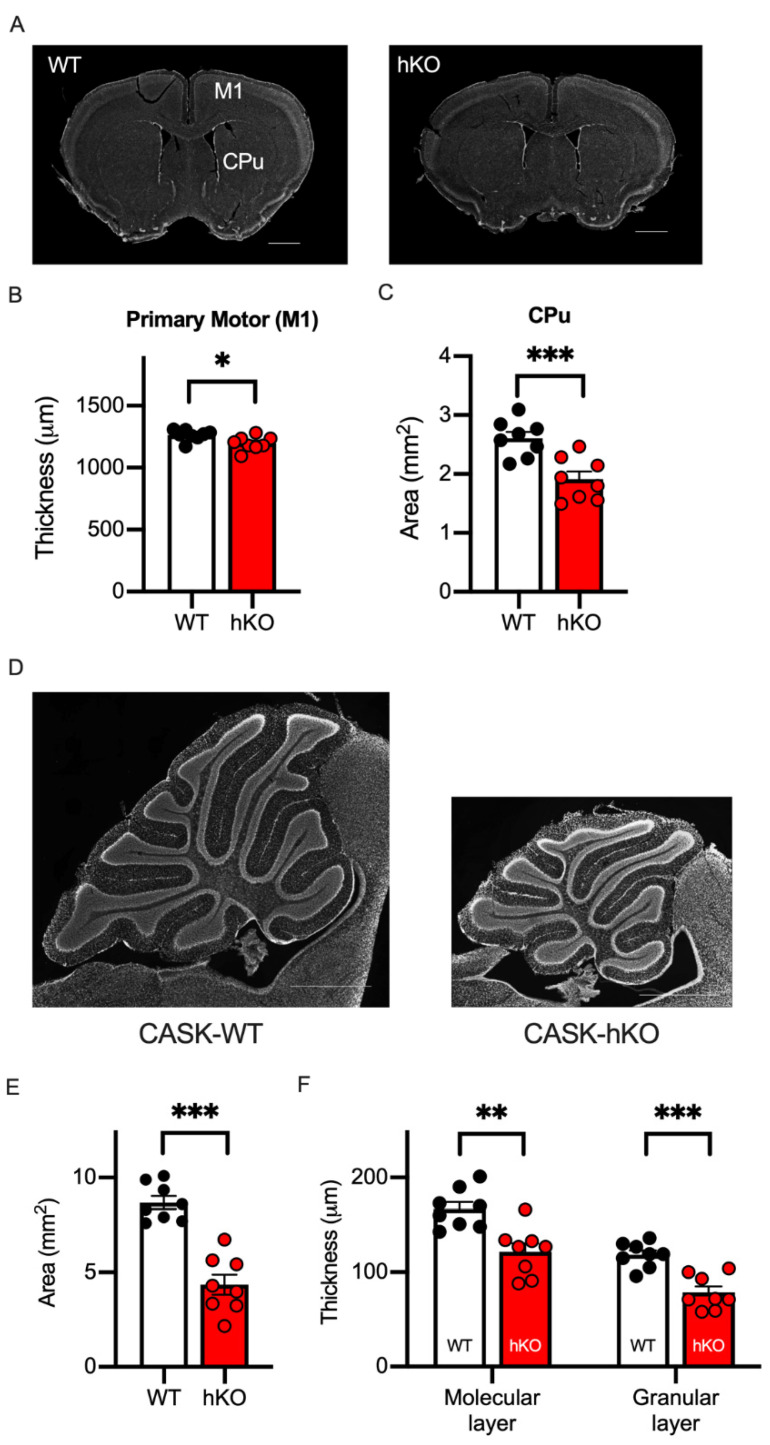
Motor associated brain regions were altered in the CASK-hKO mice. (**A**) DAPI-stained brain slices including the primary motor cortex (M1) and the caudate putamen (CPu) are shown (scale bars = 1 mm). (**B**) The thickness of M1 decreased in the CASK-hKO (heterozygote knockout) mice (n = 8) compared to the CASK-WT (wildtype) mice (n = 8). (**C**) The area of the CPu is smaller in the CASK-hKO mice than that of the CASK-WT mice. (**D**) Histological sagittal sections of the cerebellum of mice (scale bars = 1 mm). (**E**) The area of the cerebellum is smaller in the CASK-hKO mice. (**F**) The thickness of the molecular and granular layers is smaller in the CASK-hKO mice than in the CASK-WT mice. Each dot indicates the value of an individual animal. Bars and error bars indicate the mean and the standard error. Asterisks represent statistically significant differences evaluated by Student’s *t*-test (* *p* < 0.05, ** *p* < 0.01, *** *p* < 0.001).

Thus, the microcephaly and the motor deficits of CASK-related disorders were replicated in the CASK-hKO mice used in the current study. For further investigations of the neuropathological characteristics of CASK-hKO mice we prepared brain sections from both CASK-hKO and WT mice (Figure 3A) and measured various histological parameters. The thickness of the primary motor cortex (Figure 3B: 1263 ± 15.30 μm in WT vs. 1197 ± 19.96 μm in hKO, *p* = 0.0200) and the area size of the caudate putamen (Figure 3C: 2.61 ± 0.11 mm^2^ in WT vs. 1.91 ± 0.13 mm^2^, *p* = 0.0009) were reduced in the CASK-hKO mice compared with the WT mice. The cerebellum of CASK-hKO mice was significantly smaller than that of WT mice (Figure 3D,E: 8.68 ± 0.35 mm^2^ in WT vs. 4.35 ± 0.53 mm^2^ in hKO, *p* < 0.0001), and the thickness of the cerebellar layers (Figure 3F) was also altered in CASK-hKO mice, both of the molecular layer (167.0 ± 7.29 μm in WT vs. 121.5 ± 9.08 μm in hKO, *p* = 0.016) and granule cell layer (118.4 ± 4.65 μm in WT vs. 78.5 ± 6.38 μm in hKO, *p* = 0.0002). These results are consistent with the previous reports in both mice as well as human patients [11,20].

**Figure 4:**
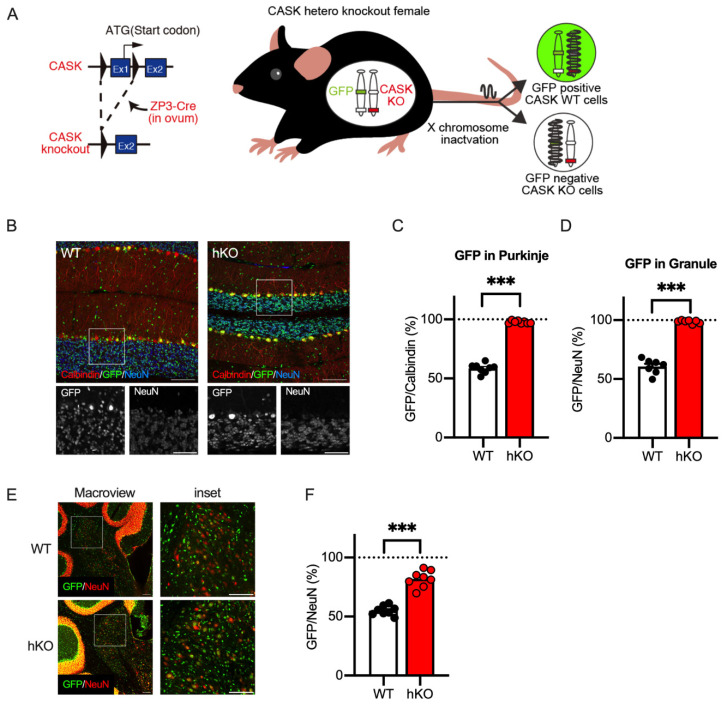
CASK-expressing neurons survived in the cerebellum of the CASK-hKO (heterozygoute knockout) female mice. (**A**) Schematic drawing of the GFP reporter mouse labeling CASK-expressing cells and CASK-deficient cells generated by X chromosome inactivation (XCI). Left: A CASK-floxed mouse [19] was crossed with a ZP3-Cre mouse, leading to the deletion of the first exon of the *CASK* gene. Right: GFP expression genetic cassette and the CASK-knockout allele were separated and the expression of GFP indicates which X chromosome was inactivated by XCI. GFP-expressing cells indicate the expression of the *CASK* gene and GFP-negative cells indicate the deficiency of the expression of the *CASK* gene. (**B**) Histological images of the cerebellum of CASK-WT (left) and CASK-hKO (right) mice. Brain sections were immunostained with antibodies against Calbindin (red) and NeuN (blue), with GFP indicating in green. Insets indicate the bottom images with a higher magnification. Scale bars are 100 μm in large images and 50 μm in small images. (**C**) The percentage of GFP-positive Purkinje cells (Calbindin-expressing) is approximately 50% in WT mice (n = 8), indicating XCI occurrs in a random fashion. On the other hand, nearly 100% of the Purkinje cells expressed GFP in hKO mice (n = 8), suggesting that CASK-deficient Purkinje cells are eliminated during the cerebellar development. (**D**) The percentage of GFP-positive cells in the NeuN-positive cells in the granular cell layer is 50% in WT (n = 8), whereas nearly 100% of GFP-NeuN double-positive cells are observed in hKO mice (n = 8). (**E**) Representative images of the deep cerebellar nucleus (DCN) from both CASK-WT and CASK-hKO mice, showing GFP in green and NeuN in red. Scale bars are 100 μm in large images and 50 μm in small images. (**F**), The percentage of GFP-positive cells over NeuN-positive cells in the DCN is higher in hKO mice (n = 8) than in WT mice (n = 8) and is significantly different. Each dot indicates the value of an individual animal. Bars and error bars indicate the mean and the standard error. Asterisks represent statistically significant differences evaluated by Student’s *t*-test (*** *p* < 0.001).

The CASK-hKO female mouse has two X chromosomes, one which is normal and the other which carries the *CASK* gene with exon 1 deleted from the genome (Figure 4A). X-chromosome inactivation is known to regulate the mesoscopic patterns of the *CASK* gene in both humans and mice [21,38,39]. To discriminate CASK-expressing and non-expressing cells in histological sections, we used a GFP reporter mouse, in which a GFP gene cassette is inserted into the HPRT gene locus [26]. As shown in Figure 4A, a GFP reporter cassette is linked to a normal *CASK* gene on an X chromosome and as such GFP-expressing cells are expected to express CASK when a CASK-heterozygote mouse is crossed with a GFP reporter mouse (CASK-hKO/HPRT-GFP). In GFP-negative cells, instead, the other X chromosome with a deficient *CASK* gene is supposed to keep active, indicating that GFP-negative cells are CASK-negative.

We investigated the cerebellar sagittal sections obtained from the CASK-hKO/HPRT-GFP females to quantify the distribution of CASK-positive and CASK-negative cells. We visualized Purkinje cells and cerebellar granule neurons with antibodies against Calbindin (CB) and NeuN, respectively, and analyzed the GFP positivity among these cerebellar cell types (Figure 4B). We found that almost all the Purkinje cells and neurons in the granular cell layers were GFP-positive (Figure 4C: 97.66 ± 0.41%; Figure 4D: 96.17 ± 0.52%) in the CASK-hKO/HPRT-GFP mice. Approximately half of the cells were GFP-positive in the WT/HPRT-GFP mice, and the percentages of GFP positivity were significantly different between the CASK-hKO and WT mice (*p* < 0.0001 in Purkinje and *p* < 0.0001 in CGC). These results indicate that almost all the Purkinje and cerebellar granule cells are CASK-positive and that CASK-negative cells were eliminated early in the cerebellar development.

We also examined the GFP positivity in the deep cerebellar nucleus (DCN, Figure 4E), the output region of the cerebellum, and observed that approximately 80% of the NeuN-positive cells were GFP-positive in CASK-hKO/HPRT-GFP, whereas half of the neurons were GFP-positive in the WT/HPRT-GFP mice (Figure 4F: 81.9 ± 2.50% of hKO vs. 55.04 ± 1.44% of WT, *p* < 0.0001). These results suggest that CASK-negative neurons were partially excluded during the cerebellar development. To evaluate the survival rate of CASK-negative cells, we calculated the probability of the survival (*P_survival_*) based on the counts of GFP-positive and GFP-negative cells. *P_survival_* of the Purkinje cells was 71.4 ± 4.3% in WT/HPRT-GFP and 2.4 ± 0.4% in the CASK-hKO/HPRT-GFP mice. A similar difference was observed in the case of CGCs: 66.7 ± 7.0% in WT and 1.4 ± 0.5% in CASK-hKO. *P_survival_* of the neurons in the DCN of CASK-hKO/HPRT-GFP was 22.9 ± 3.9%, which is still lower than that of the WT/HPRT-GFP mice (82.6 ± 4.8%, *p* < 0.0001). These results suggest that neurons in the DCN are eliminated partially during the cerebellar development.

**Figure 5:**
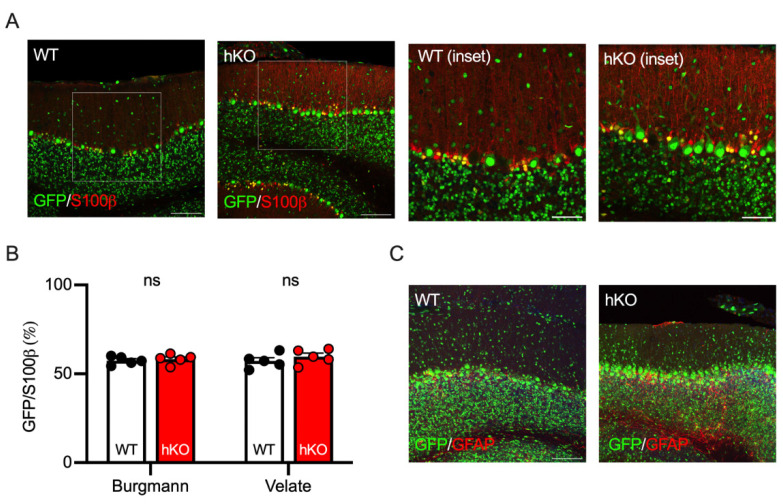
GFP expression patterns of cerebellar astrocytes were not altered between CASK-WT and hKO mice. (**A**) Histological images of S100β protein (red) expressed in Burgmann astrocytes in the Purkinje cell layer and in velate astrocytes in the granular cell layer of the cerebellum. GFP (green) represents the activated X-chromosome by XCI. Insets indicate the areas of enlarged pictures. (**B**) The percentages of GFP-positive cells in the glial cells were nearly 50% both in Burgmann and velate astrocytes, which were not significantly different between WT and hKO mice. Approximately half of the S100β-positive cells expressed GFP in both WT and hKO mice. (**C**) Histological images of GFAP (green) immunohistochemistry in CASK-WT and hKO mice. Scale bars are 100 μm in large images and 50 μm in insets.

The structure and function of the cerebellum are maintained not only by neurons but also by glial cells. Two types of astroglia exist in the cerebellum [40,41]. One is Bergmann glia, which is localized along the Purkinje cell layer, extends its fibers into the molecular layer, and plays a role in maintaining the concentrations of extrasynaptic glutamate and potassium [42]. The other is velate astrocyte, which is located in the granule cell layer and is involved in the cerebellar granule glomeruli where mossy fibers form synapses on cerebellar granule cells [43]. Both glial cell-types were visualized with an antibody against S100β protein and the GFP positivity was determined on the cerebellar section from adult animals (Figure 5A). As observed in cerebellar neurons (Figure 4), approximately half of Bergmann (57.5 ± 1.0%) and velate (57.3 ± 1.8%) glia were GFP-positive in WT mice (Figure 5B). The ratio of the GFP-positive cells was not altered in CASK-hKO/HPRT-GFP mice (Figure 5B: 58.3 ± 1.2% in Bergmann glia, 59.7 ± 1.9% in velate glia), indicating no genotype-specific difference. We also stained these glial cells with another antibody against glial fibrillary acidic protein (GFAP) and observed that the number of the glial cells was not different between WT and CASK-hKO. Furthermore, we occasionally observed glia cells with a strong expression of GFAP in patches in the cerebellum of CASK-hKO mice (Figure 5C). The strong and patchy expression of S100b was not obvious, probably because S100b staining was clearer in Bergmann glia than in velate glia (Figure 5A).

**Figure 6:**
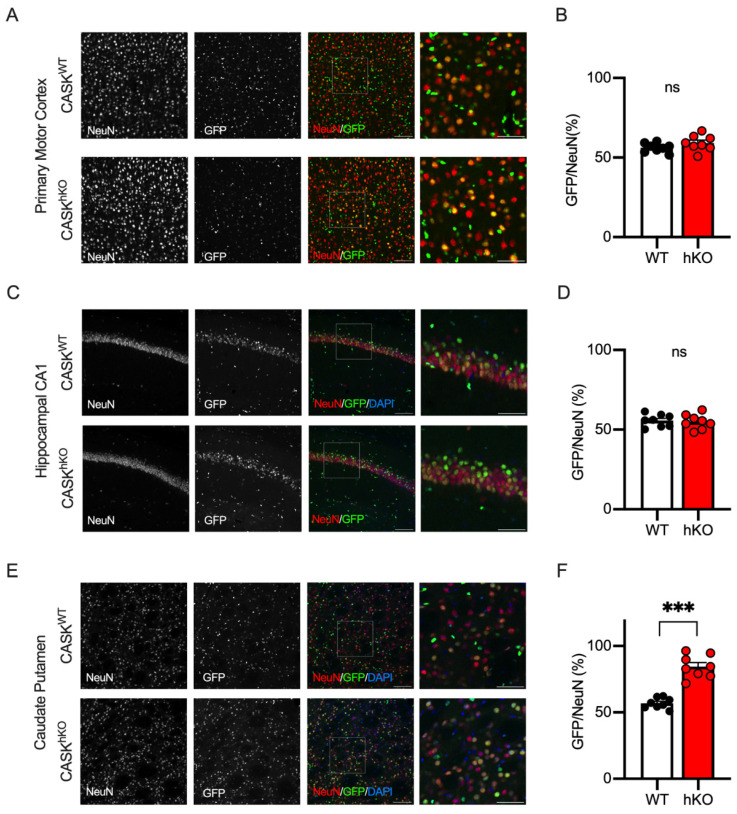
GFP-expression exhibited distinct patterns in other motor-associated brain regions. (**A**) Histological images of layer 2/3 of the M1. NeuN staining (leftmost), GFP-expression (the second from the left), and overlapped images (the third from the left) in WT (wildtype, CASK^WT^, top) and hKO (heterozygote knockout, CASK^hKO^, bottom) mice are shown. NeuN and GFP are colored in red and green, respectively. The rightmost images are enlargements of the insets on the overlapped images. (**B**) Approximately 50% of NeuN-positive cells expressed GFP in the layer 2/3 of the M1 in WT (n = 8) and hKO (n = 8) mice, without a significant difference (ns: not significantly different). (**C**) Histological images of hippocampal CA1 region. NeuN staining (leftmost), GFP expression (the second from the left), and overlapped images (the third from the left) in WT (top) and hKO (bottom) mice were shown. NeuN and GFP were colored in red and green, respectively. The rightmost images are enlargements of the insets on the overlapped images. (**D**) Approximately 50% of NeuN-positive cells express GFP in the stratum pyramidale of the hippocampal CA1 region in WT and hKO mice, without a significant difference. (**E**) Histological images of the caudate putamen of the striatum in WT and hKO mice are shown. (**F**) Nearly 80% of the NeuN-positive cells express GFP in hKO mice, whereas 50% do in WT mice. Scale bars are 100 μm (left three images of (**A**,**C**,**E**)) or 50 μm (right image of (**A**,**C**,**E**)). Asterisks represent statistically significant differences evaluated by Student’s *t*-test (*** *p* < 0.001).

We previously reported that the CASK positivity of the neurons in the neocortex and the hippocampus of CASK-hKO mice was approximately 50% using single-cell RT-PCR [21], suggesting that CASK deficiency may not affect the survival of the neocortical and hippocampal neurons. To examine this hypothesis histologically, we examined GFP positivity in the neocortex (the primary motor cortex, Figure 6A,B) and the hippocampus (CA1, Figure 6C,D). We found that the GFP positivity of neocortical and hippocampal neurons was 59.2 ± 1.7% and 55.0 ± 1.6% in CASK-hKO mice, respectively, which were not altered compared with the WT mice (56.2 ± 1.0% in the neocortex and 55.7 ± 1.4% in the hippocampus). These results indicate that the survival of these neurons may not be affected, as we observed in the previous study. We further examined the GFP positivity in another motor-associated brain region, the caudate putamen of the striatum (Figure 6E). Approximately 84.6 ± 3.0% of the NeuN-positive cells in the caudate putamen of CASK-hKO mice were GFP-positive, whereas 56.9 ± 1.4% of the cells were positive in WT mice (Figure 6F), which was significantly different (*p* < 0.0001, *t*-test). The survival probability of striatal neurons was 19.3 ± 4.3% in hKO and 76.5 ± 4.4% in WT (*p* < 0.0001, *t*-test), which was significantly altered.

**Figure 7:**
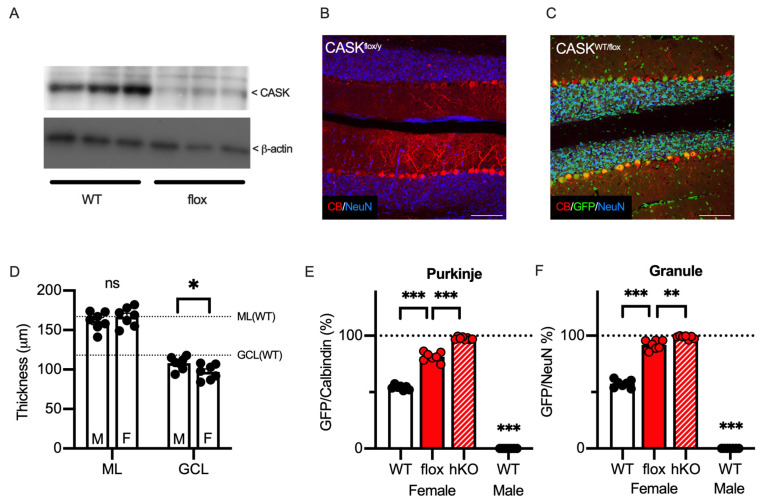
Cerebellar hypoplasia was observed in a hypomorphic CASK mouse. (**A**) Immunoblot of CASK protein (top) and beta-Actin (bottom) in the cerebellum of CASK-hypomorphic (flox-CASK, fCASK, n = 3) and WT (n = 3) adult mice. The expression of CASK protein is lower in the fCASK male mice than in the CASK-WT male mice. The whole blotting images are seen in Supplementary Figure S2. (**B**) Histological image shows that Purkinje (Calbindin, red) cells remain in the cerebellum of the hemizygote CASK-floxed male mouse. Neurons were visualized with NeuN (blue). (**C**) Histological images of the cerebellum from heterozygous CASK-floxed female mice (CASK^WT/flox^) with an HPRT-GFP allele. GFP, Calbindin, and NeuN signals are shown in green, red, and blue, respectively. GFP-negative cells represent CASK-hypomorphic cells in which an X chromosome with floxed CASK is activated. (**D**) Thicknesses of molecular layer (ML) and granular cell layer (GCL) of CASK-floxed-hemizygote males (M, n = 7) and -heterozygote females (F, n = 7) are shown on the graph. Dashed lines indicate the thickness of the molecular layer (top) and the granule cell layer (bottom), which are analyzed in Figure 3F. (**E**) A total of 81.4 ± 1.53% of Purkinje cells were GFP-positive in the CASK-hypomorphic female mice (n = 7), which was significantly higher than in CASK WT female mice (n = 7). (**F**) A total of 91.9 ± 1.56% of granule cells were GFP-positive in the CASK-hypomorphic female mice (n = 7), which was significantly higher than in CASK WT female mice (n = 7). The percentage of GFP cells was significantly higher in the CASK hKO female mice (n = 7) and no GFP cells were detected in WT male mice (n = 7). Scale bars are 100 μm in (**B**,**C**). Asterisks represent statistically significant differences evaluated by Student’s *t*-test (* *p* < 0.05, ** *p* < 0.01, *** *p* < 0.001, ns: not significantly different).

The results thus far indicate that *CASK* gene expression regulates neuronal survival in several brain regions, with particularly strong effects in the cerebellar cortex. Najm et al. originally reported hypoplasia of the cerebellar cortex of the male floxed CASK (fCASK)-hemizygote mice [11], which were known to express only 30% of the CASK protein compared of the WT mice [19]. These findings give rise to a hypothesis that a low expression of the CASK protein may affect the survival of the cerebellar Purkinje and/or granule cells. We confirmed the lower expression level of the CASK protein in the cerebellum of male fCASK-hemizygote mice (52.86 ± 8.76% compared to WT, Figure 7A). Even though the expression level of the CASK protein was smaller, we observed aligned Purkinje cells and a layer of granule cells in the cerebellar cortex of male floxed mice (Figure 7B). We also crossed the fCASK mice with the HPRT-GFP mice and obtained female fCASK/HPRT-GFP-heterozygote mice, whose cerebellar cortex resembled that of WT mice (Figure 7C). We measured the thickness of the molecular and granule cell layers of the cerebellum in both male and female mice (Figure 7D) and observed that the granule cell layer (GCL) was significantly thinner in heterozygote females than in hemizygote males (108.1 ± 3.1 μm in fCASK-hemizygote males vs. 97.0 ± 3.6 μm in fCASK-heterozygote females, *p* = 0.0383). The granule cell layer was also thinner in fCASK-heterozygote females than in control female mice (*p* = 0.00359). The GFP positivity of the Purkinje cells in the cerebellum of the female fCASK/HPRT-GFP mice was 81.4 ± 1.5%, whereas it was 54.2 ± 0.8% in WT/HPRT-GFP females (Figure 7E, *p* < 0.0001). The survival probability of the Purkinje cells was calculated as 23.2 ± 2.4% in fCASK/HPRT-GFP and 84.9 ± 2.6% in WT/HPRT-GFP, which was significantly different (*p* < 0.0001). The GFP positivity of the cerebellar granule cells was 91.9 ± 1.6% in fCASK/HPRT-GFP, significantly higher than the 57.4 ± 1.3% seen in WT/HPRT-GFP (Figure 7F). The survival possibility of the cerebellar granule cells was significantly lower in fCASK/HPRT mice (8.96 ± 1.9% in fCASK/HPRT-GFP vs. 74.8 ± 1.9% in WT/HPRT-GFP, *p* < 0.0001). These results suggest that a mixture of CASK-expressing and CASK-deficient neurons have a certain impact on the survival of cerebellar neurons. Furthermore, in comparison with CASK-hKO mice, the CASK-hypomorph female mice showed a lower ratio of GFP-positive Purkinje and granule cells (Figure 7E,F), suggesting that the expression dose of CASK protein is a key factor in the survival of cerebellar neurons.

**Figure 8:**
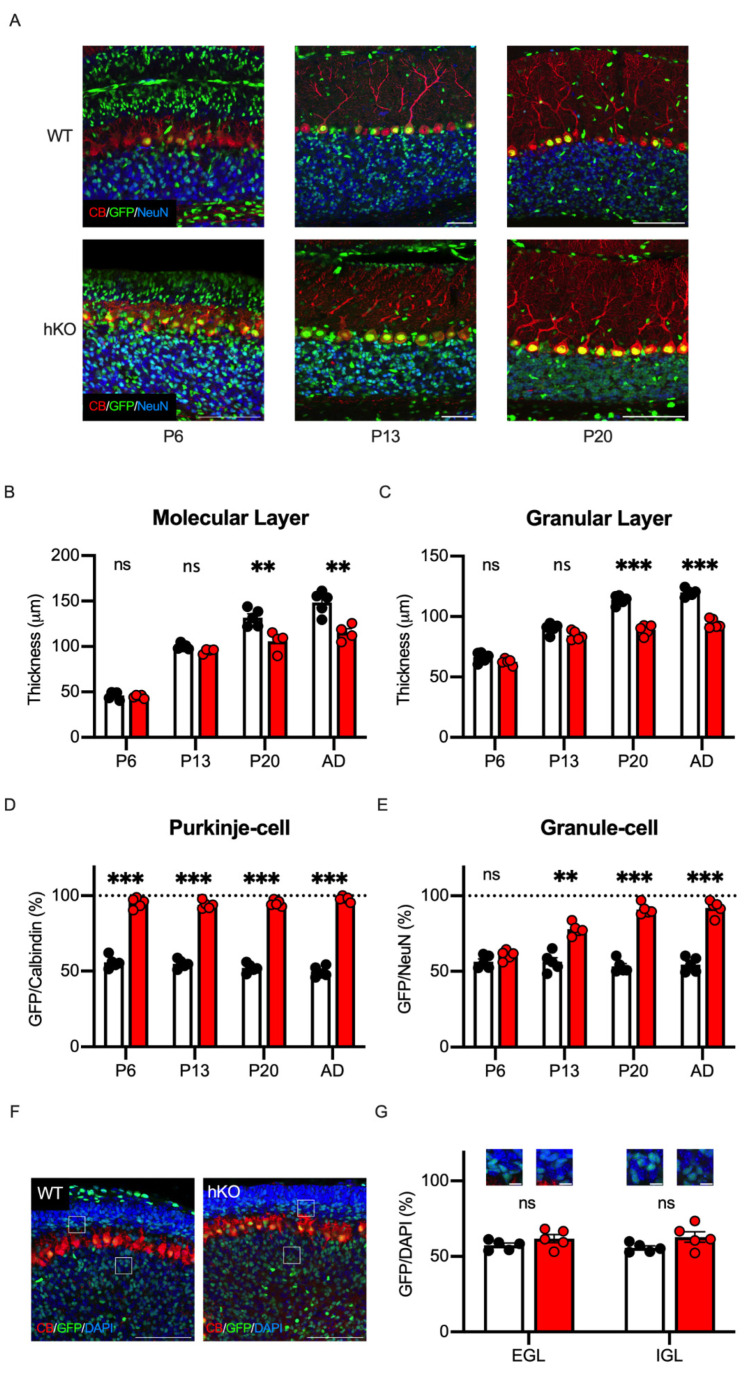
The survivals of Purkinje and Granule cells were differentially regulated during the postnatal development. (**A**) Histological images of the cerebellum at different ages of mice are shown. Rightmost pictures are those obtained from mice at postnatal day 6 (P6, n = 5 in WT; n = 5 in hKO). Middle pictures are those from mice at postnatal day 13 (P13, n = 5 in WT; n = 4 in hKO). Leftmost pictures are those from mice at postnatal day 20 (P20, n = 5 in WT; n = 4 in hKO). GFP, Calbindin, and NeuN signals are shown in green, red, and blue, respectively. Scale bars indicate 100 μm. Note that the length of the scale bars is different in each picture. (**B**) The molecular layer thickens more slowly in hKO mice (red) compared to the WT mice (white/black), especially at P20 and in adulthood (n = 5 in WT and n = 4 in hKO). The thickness was not significantly different at young stages (ns: not significantly different). (**C**) The difference in the thickness of the granular layer becomes significant after P13. (**D**) Nearly 100% of Purkinje cells are GFP-positive as early as P6, which is when Purkinje cells finish their positioning in the cerebellum. (**E**) The percentage of GFP-positive granule cells increases during the postnatal development in hKO mice, whereas the percentage is unchanged in WT mice. (**F**) Histological images of external and internal granular cell layers of mice at P6 are shown. GFP is shown in green and DAPI is in blue. Scale bars indicate 100 μm. (**G**) The percentages of GFP positivity on DAPI-stained cells in the external granular layer (EGL), the intermediate molecular layer (ML), and the internal granular layer (IGL) are presented. Scale bars indicate 20 μm. Asterisks represent statistically significant differences evaluated by Student’s *t*-test (** *p* < 0.01, *** *p* < 0.001).

We previously reported that CASK developmentally regulated the survival of the cerebellar granule cells in vitro [22]. To further elucidate the developmental process of the cerebellar hypoplasia in vivo, we investigated the developmental changes in the CASK-deficient cerebellar cells by analyzing the cerebellar sections at several postnatal stages. As shown in Figure 8A, we prepared cerebellar sections at postnatal day 6 (P6, the age when granule cells begin migration), postnatal day 13 (P13, the age when migration ends), and postnatal day 20 (P20, the age when cerebellar cortex maturation ends) [44]. At the postnatal stages, the molecular and granule cell layers kept thickening toward adulthood in WT mice, but the cerebellar layers of the CASK-hKO mice did not grow after 3 weeks-old (Figure 8B,C). We next investigated the GFP positivity of Purkinje cells at these developmental stages and found that the GFP positivity was nearly 100% at as young as P6 (Figure 8D), indicating that CASK deficiency eliminates Purkinje cells or the progenitor cells of Purkinje cells before P6. On the other hand, the GFP positivity of the cerebellar granule cells at P6 was approximately 50% in both WT and CASK-hKO mice. The GFP positivity thereafter increased along the developmental stages in CASK-hKO mice, whereas it was about 50% at all the postnatal stages of WT mice (Figure 8E). We visualized the neurons with an antibody against NeuN, but the neural progenitors are known to be NeuN-negative (Figure 8A). Therefore, we stained the brain sections with DAPI to visualize the external and internal granular cell layers (Figure 8F). The GFP positivity of the external and internal granule cells at P6 was close to 50% (Figure 8G), suggesting that CASK does not affect the survival of immature granule cells and/or neural progenitors of cerebellar granule cells.

**Figure 9:**
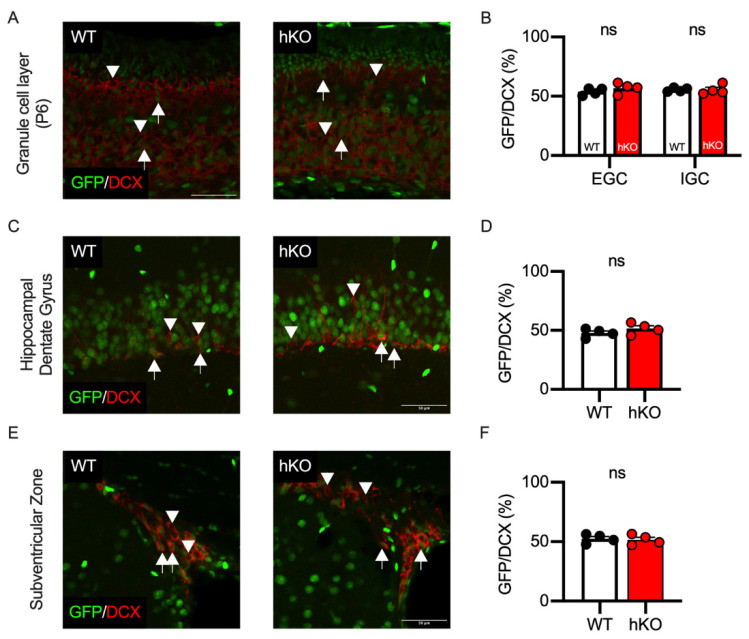
GFP positivity of DCX-positive cells was not affected in CASK-hKO (heterozygote knockout) mice. (**A**) Histological images of the cerebellum at P6 are shown. GFP and doublecortin (DCX) immunosignals are indicated in green and red, respectively. Arrows in the images indicate GFP/DCX double-positive cells and arrowheads in the images indicate DCX single-positive cells. The image of WT (wildtype) mice is on the right and that of hKO is on the left. (**B**) Quantification of GFP positivity shows no significant difference between WT and hKO mice. (**C**) Histological images of hippocampal dentate gyrus in adult WT (left) and hKO (right) mice. GFP and DCX are indicated in green and red, respectively. Arrows indicate GFP/DCX double-positive cells and arrowheads indicate DCX single-positive cells. (**D**) Nearly half of the DCX-positive cells express GFP in the hippocampal dentate gyrus in both WT and hKO mice. (**E**) Histological images of the subventricular zone of adult WT (left) and hKO (right) mice. GFP and DCX are indicated in green and red, respectively. Arrows indicate GFP/DCX double-positive cells and arrowheads indicate DCX single-positive cells. (**F**) GFP positivity on the DCX-positive cells is not different between WT and hKO mice. ns represents that no statistical significance was detected by by Student’s *t*-test (*p* > 0.1).

To investigate the effects of CASK on the proliferation of neural progenitors, we stained the brain tissues with an antibody against doublecortin (DCX), a marker of immature neurons. We found that about half of the DCX-positive EGCs and IGCs were GFP-positive in both WT and CASK-hKO mice (Figure 9A,B). In the cerebellar development, granular cell progenitors are divided in the external granular layer and migrate to the internal granular layer [44]. Thus, CASK expression may not affect the proliferation and migration of the cerebellar granule cells, rather it may affect the survival of cerebellar granule cells, which is consistent with our previous result. We further investigated the effect of CASK on the cell proliferation using immunohistochemistry against DCX. There are two brain regions known to maintain neurogenesis in adulthood, which are the dentate gyrus of the hippocampus (Figure 9C,D) and the subventricular zone (Figure 9E,F). The percentage of the GFP-positive cells among the DCX-positive cells was nearly 50% in the hippocampus (Figure 9D) and the subventricular zone (Figure 9F), indicating that CASK may not affect neuronal proliferation in these regions either.

## 4. Discussion

Heterozygous nonsense and missense mutations in CASK cause microcephaly with pontine and cerebellar hypoplasia in humans. CASK mutations are also known to cause other neurological phenotypes such as intellectual disability, developmental delay, and ophthalmological abnormalities [11,15,45,46]. CASK-knockout mice have been studied and have successfully reproduced the pathological phenotypes of CASK-related disorders [11,21,47]. In this study, we specifically focused on the cerebellar structure, which commonly shows severe phenotypic changes in various human cases. As we and other groups have previously observed, the cerebellum of CASK-heterozygous-knockout mice is small (Figure 1 and Figure 3). Motor deficits have been reported as a common developmental phenotype of CASK-related disorders [8]. We examined the motor abilities of the mice using several behavioral tests and observed that CASK-hKO mice showed changes not only in cerebellum-dependent motor abilities but also in general motor abilities related to neuromuscular function. Firstly, we observed a decrease in the distance traveled, which could indicate a motor impairment or an increase in anxiety levels [48]. In addition, the mice did not show a tendency to escape from the center of the exercise area, suggesting that the decrease in motor ability of the mice may be due to a decrease in the function of motor-related brain regions such as the cerebellum and striatum. The wire hang test and the rotarod test were used to evaluate motor impairment models in animals, such as cerebellar ataxia and Parkinson’s disease. These tests did not rule out the possibility that brain regions other than the cerebellum were involved. We also found changes in the mosaic distribution of cells expressing CASK and cells lacking CASK in the striatum. To clarify the involvement of the cerebellum, striatum, and other brain regions, it is necessary to analyze conditional knockout mice in which the *CASK* gene is knocked out in a brain region-specific manner [20].

X-chromosome genes are known to be regulated by XCI, which results in a mosaic distribution of neurons in the brain. The *CASK* gene has been reported to be regulated by XCI both in humans [38,39] and mice [21]. The stochastic distribution of CASK-expressing cells and non-expressing cells was observed in the early stages of postnatal development, even in cerebellar granule cells where a biased distribution of cells expressing CASK is observed in adults. The proportion of cerebellar granule cells expressing GFP increased with postnatal development, suggesting that cerebellar granule cells that do not express the *CASK* gene were eliminated. We previously reported that cerebellar granule cells undergo apoptosis when the *CASK* gene is knocked out in vitro. These results suggest that the cell-autonomous reduction in cerebellar granule cells leads to the development of an incomplete cerebellar granule cell layer. On the other hand, almost all Purkinje cells were already GFP-positive at P6. Although the developmental process of Purkinje cells differs from that of cerebellar granule cells, the survival of Purkinje cells is also thought to be regulated in a cell-autonomous manner, dependent on *CASK* gene expression.

The finding that the cerebellum is smaller in CASK-hKO mice is consistent with previous reports [20]. This report also analyzed the effects of deleting the *CASK* gene in each cell subtype by combining cerebellar granule cell- and Purkinje cell-specific Cre mice. The authors reported that the brain morphology and motor function of these mice were not different from those of fCASK mice. In our study, we observed that floxed mice exhibited reduced CASK protein levels and atrophic cerebellums. Interestingly, the cerebellar atrophy was more severe in female heterozygous mice, which had a mixture of cells with normal CASK expression and cells with reduced CASK expression, than in male hemizygous mice, which had reduced CASK expression in all cerebellar cells. Taken together, it is suggested that a decrease in *CASK* gene expression causes cerebellar cell loss in a cell-autonomous manner. However, in a situation where normal cells and CASK-deficient cells coexist, the pathology manifests itself in a more serious form due to a competitive mechanism.

Unlike the cerebellar cortex, the percentage of GFP-expressing cells in the DCN and caudate nucleus did not reach 100%, and was instead around 80%. The calculated cell survival rate was also different as in the cerebellar cortex the survival rate was almost 0%, whereas in the brain region approximately 20% of the cells remained. The partial survival of neurons suggests two possibilities. One possibility is that factors other than CASK are involved in the survival of these cells, like cerebellar granule cells [22]. The other possibility is that only the different neuronal subtypes present in these neural regions are maintained in a CASK-dependent manner. The DCN contains neurons that project to brain regions such as the thalamus and ventral tegmental area (VTA), which are involved in motor control and coordination [49]. The caudate nucleus of the striatum contains two types of neurons that make up neural circuits that express different dopamine receptors and project differently [50,51]. In the future, it is hoped that the impact of *CASK* gene expression on the functional structure of neural circuits will become clear by knocking out the *CASK* gene specifically in these neuronal subtypes.

In non-neuronal cells it has been reported that the *CASK* gene is involved in the proliferation and survival of neurons. We analyzed the expression pattern of CASK in DCX-positive cells, which are markers of immature neurons. The results suggest that *CASK* gene expression does not affect cell division in cerebellar granule cells in adulthood, as well as in juvenile cerebellar granule cells. In addition, it is known that cerebellar granule cells migrate from the external granule layer to the internal granule layer, but the proportion of CASK expression was the same at around 50% in the external and internal granule layers. These results suggest that CASK expression does not affect neuronal migration.

A technical limitation of this study is the accuracy of the quantification of GFP cells. It is known that GFP signals are similar to autofluorescence signals, and in this study GFP cells were likely over-counted due to autofluorescence [52]. Most tissue samples from CASK-WT/HPRT-GFP mice showed over 50% GFP positivity, which may have impacted our assessment of cell viability. For example, the survival rate of cells in the DCN was evaluated to be 20% viable, but the actual viability may be higher than the calculated viability. In order to overcome this difficulty we attempted immunohistochemistry to detect CASK protein, but even when using available antibodies CASK protein in brain tissue was below the detection limit. As shown in the Allen brain atlas, CASK mRNA is also below the detection limit by in situ hybridization on the Allen brain atlas (Experiment #69134622 & #70918868) [53]. Based on the above, the use of HPRT-GFP mice is currently considered to be the optimal method for evaluating XCI, although it still has inherent technical constraints.

## 5. Conclusions

This study analyzed the microcephaly of CASK-related disorders, which are caused by mutations in the X-linked gene CASK, using histopathological methods. In model mice that mimic the condition in female patients, who make up the majority of CASK-related-disorders patients, cerebellar hypoplasia was observed. Both CASK-expressing cells and non-expressing cells were observed only in the cerebellum, with a notable developmental change in the proportion of these cells. Specifically, cerebellar granule cells lacking CASK were eliminated over time. In addition, this cell loss was more pronounced in the presence of both CASK-expressing cells and non-expressing cells. Thus, it is thought that in female patients with CASK-related disorders, cerebellar hypoplasia develops due to a combination of cell-autonomous effects and the competitive cell elimination of cerebellar neurons.

## Figures and Tables

**Table 1:** List of PCR primers.

	Annealing (°C)	Direction	Sequence 5′ to 3′
CASK floxed (fCASK)	62	forward	CTTGGTCGCAGCTTGGGAGTA
		reverse	GGACTAACCCTCCTCCCTTTC
ZP3-Cre	62	forward	GAAGATCTTCCAATTTACTGACCGTACAC
		reverse	CCATGAGTGAACGAACCTGGTCGA
Cask-KO	62	forward	CTTGGTCGCAGCTTGGGAGTA
		reverse	TTTGGGGACTAGATGGGTGTGGTG
HPRT-GFP	60	forward	GAACCTATTATGCTGGCTAGTCAC
		reverse	CACCAGTGAAGAGCACTGGATGC

**Table 2:** List of primary antibodies for immunohistochemistry.

**Table**	**Host**	**Dilution**	**Provider**	**RRID**
Calbindin	Rabbit	1:2000	Frontier Institute, Sapporo, Japan	AB_2571568
GFP	Chicken	1:1000	Aves labs, Davis, CA, USA	AB_10000240
NeuN	Mouse	1:500	Millipore, Burlington, MA, USA	AB_2298772
S100β	Rabbit	1:1000	Frontier Institute, Sapporo, Japan	AB_2725784
GFAP	Rabbit	1:1000	Frontier Institute, Sapporo, Japan	AB_2571707
DCX	Rabbit	1:1000	Abcam, Cambridge, UK	AB_732011

## Data Availability

Codes and materials will be available on request.

## Supplementary Materials

The following supporting information can be downloaded at: https://www.mdpi.com/article/10.3390/cells14100735/s1, Figure S1: Schematic explanation for the estimation of the possibility of cell survival. Figure S2: Gel images of the immunoblot. Word file of legends of suppmentary figures is available.
