# Supplementary material for: The Competitive Loss of Cerebellar Granule and Purkinje Cells Driven by X-Linked Mosaicism in a Female Mouse Model of CASK-Related Disorders
Source: Cells. 2025 May 17;14(10):735. doi: 10.3390/cells14100735 (PMC12109812; doi:10.3390/cells14100735)
Supplement: Supplementary file 1 [file cells-14-00735-s001.zip › Supplementary_Figure_Legend.pdf]

Supplementary Figure S1. Schematic explanation for the estimation of the possibility of cell survival. An example shows that the proportion of GFP-expressing cells on the histological sample is 75%. We set assumptions that the same number of GFP-positive and GFP-negative cells after the cell division, and that 100% of the GFP-positive cells survive until the adulthood. Under these assumptions, the survival probability ($P_{Survival}$) of GFP-negative cells is calculated to be 33%.

Supplementary Figure S2

Gel images of the immunoblot detecting CASK (left) and beta-actin (right) are shown. Cerebellar homogenates from three WT mice were loaded on the three left lanes and those from three hemizygote Cask-floxed mice (flox) were on the three right lanes. Rectangles indicate the images shown in Figure 7A.
