# Supplementary figures and images for: The Competitive Loss of Cerebellar Granule and Purkinje Cells Driven by X-Linked Mosaicism in a Female Mouse Model of CASK-Related Disorders
Source: Cells. 2025 May 17;14(10):735. doi: 10.3390/cells14100735 (PMC12109812; doi:10.3390/cells14100735)

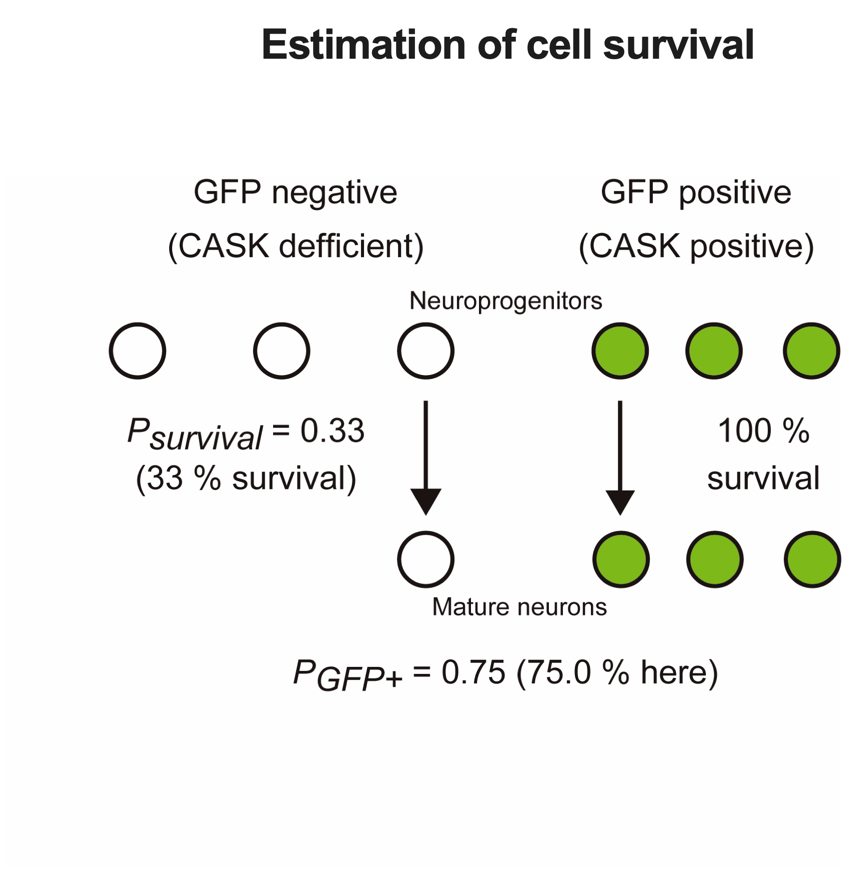

Supplement: Supplementary file 1 [file cells-14-00735-s001.zip › Supplementary FigS1.tiff]

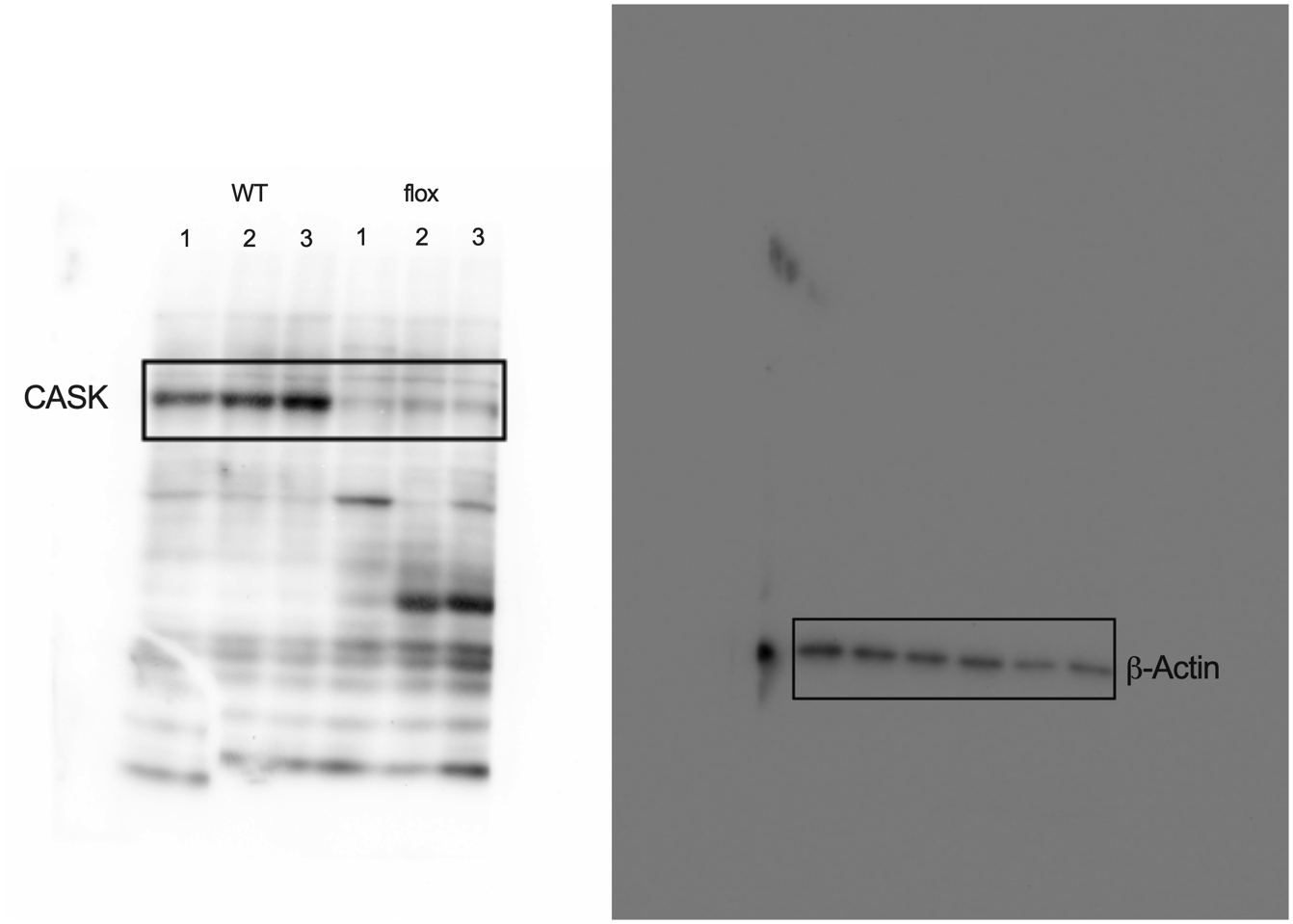

Supplement: Supplementary file 1 [file cells-14-00735-s001.zip › Supplementary FigS2.tiff]
